# Sub-Block Urban Function Recognition with the Integration of Multi-Source Data

**DOI:** 10.3390/s22207862

**Published:** 2022-10-16

**Authors:** Baihua Liu, Yingbin Deng, Xin Li, Miao Li, Wenlong Jing, Ji Yang, Zhehua Chen, Tao Liu

**Affiliations:** 1 College of Geographical Science, Harbin Normal University, Harbin 150025, China; 2 Guangdong Open Laboratory of Geospatial Information Technology and Application, Laboratory of Guangdong for Utilization of Remote Sensing and Geographical Information System, Guangzhou Institute of Geography, Guangdong Academy of Sciences, Guangzhou 510070, China; 3 Southern Marine Science and Engineering Guangdong Laboratory (Guangzhou), Guangzhou 511485, China; 4 Guangdong Provincial Institute of Land Surveying & Planning, Guangzhou 510075, China

**Keywords:** multi-source data, urban functional area, sub-block, random forest

## Abstract

The recognition of urban functional areas (UFAs) is of great significance for the understanding of urban structures and urban planning. Due to the limitation of data sources, early research was characterized by problems such as singular data, incomplete results, and inadequate consideration of the socioeconomic environment. The development of multi-source big data brings new opportunities for dynamic recognition of UFAs. In this study, a sub-block function recognition framework that integrates multi-feature information from building footprints, point-of-interest (POI) data, and Landsat images is proposed to classify UFAs at the sub-block level using a random forest model. The recognition accuracies of single- and mixed-function areas in the core urban area of Guangzhou, China, obtained by this framework are found to be significantly higher than those of other methods. The overall accuracy (OA) of single-function areas is 82%, which is 8–36% higher than that of other models. The research conclusions show that the introduction of the three-dimensional (3D) features of buildings and finer land cover features can improve the recognition accuracy of UFAs. The proposed method that uses open access data and achieves comprehensive results provides a more practical solution for the recognition of UFAs.

## 1. Introduction

Cities are composed of various functional areas that can effectively perform specific urban functions, such as residential, commercial, and industrial areas. The functional differences in these regions are the result of the combined effects of different socioeconomic activities. Today, the rapid development of urbanization has led to drastic changes in the functional layout of cities; this has disrupted the original interconnected and stable internal spatial structure of cities, and has also caused various problems in urban development [1,2]. Urban functional areas (UFAs), as the basic unit of urban planning and management, are affected not only by planning policies, but also by the way of civil life, and change with urban development. As the distribution of UFAs is related to human activities, it is of great significance to divide UFAs and understand their spatial distribution and characteristics for the coordination of human–land relationships, urban planning management, environmental change monitoring, and resource allocation optimization [3,4,5,6].

Traditionally, UFAs recognition has mainly relied on qualitative methods such as statistical investigation and expert judgment [7,8]. However, the data are difficult to obtain, and the investigation and analysis require large amounts of time and labor costs. Moreover, the reliability of the results is also characterized by problems such as strong subjectivity and poor timeliness [9,10].

With the development of remote sensing technology, scholars have found that remote sensing images can effectively extract ground object information [11]. The dynamic monitoring of urban land-use changes can be realized by analyzing remote sensing images in different periods; thus, remote sensing images are widely used in land-use and land cover research [12,13]. Many studies have explored the possibility of using remote sensing images to classify urban land cover and functional areas [14,15]. These methods display remarkable advantages in extracting the physical properties of ground objects. However, the function of urban land is not only related to the physical properties of land blocks, but is also affected by human activity, and it is difficult to determine the socioeconomic characteristics of different regions by relying solely on remote sensing image information [16].

In recent years, social sensing data, such as point-of-interest (POI) data, travel trajectory data, and social media check-in data, have provided new opportunities for research on urban internal regional functions [17,18,19]. These emerging geospatial data are rich in the semantic features of human mobility and socioeconomic activities, thus contributing to a better understanding of the functional interaction patterns and social structures of urban environments [20]. Among them, POI data are favored because of the close correlation between category labels and land-use types and the relative ease of data acquisition [21]. However, the performance of methods that use only POI data is affected by the data coverage. For underdeveloped areas and privacy-conscious residential areas, the function classification of these areas is difficult to predict accurately due to the lack of POI data [22].

The integration of multi-source data can compensate for the shortcomings of single-source data in the recognition of UFAs, and can more accurately reveal the spatiotemporal characteristics of UFAs; thus, this has become an important direction in the achievement of the refined study of UFAs [23]. For example, functional area recognition has been performed based on spectral features, landscape features, and latent semantic features via the fusion of remote sensing images and social sensing data [24]. The maximum expectation algorithm has been used to extract the time-series features of trajectory data, and the Delphi method has been used to mine the semantic information of the density and category of POI data, combined with their analysis for realizing functional area identification [25]. The dominant function of each area has been identified based on the features of different functional types extracted from POIs and the distribution of the check-in intensities of different functional types estimated by the kernel density method [26]. The information mapped by different data is different, and the fusion analysis of multi-source data can more comprehensively reflect the regional features of ground objects, the characteristics of human activity, and the spatiotemporal changes in social information.

In previous studies, the fusion of remote sensing data and social sensing data, as well as the fusion of social sensing data with other social sensing data, has been common [27,28,29,30]. However, the joint use of these types of data with building information has been easily ignored in feature mining, and most researchers have used the spectral, texture, and spatial features of images without considering the relationship between different land cover features and UFAs [31]. In addition, the results of the existing studies are generally divided into several single-function areas, and a discussion of mixed-function areas is lacking [32]. As the main setting of human activities, buildings can reflect the urban form and regional functions to a certain extent, and there are obvious differences in the land cover composition within different functional areas; thus, it is important to understand the differences in the building morphology and finer land cover features of urban areas for function classification [33]. Moreover, in complex and heterogeneous urban areas, the function of areas is often not completely pure, and it would be more in line with the real situation to consider a functional mix.

To overcome these existing problems, this study proposes a functional area recognition framework that integrates multi-feature information and can successfully find single- and mixed-function areas in the study area. By deeply mining the multi-feature information of building footprints, POIs, and remote sensing images, the fine recognition of functional areas at the sub-block level is achieved by using random forest classifiers. The contributions of this research are mainly divided into the following three points. (1) The introduction of the three-dimensional (3D) features of buildings and finer land cover features of images effectively improves the classification accuracy of UFAs. (2) By using open access multi-source data, the mapping of UFAs with coexisting single- and mixed-function areas is realized. (3) The accuracies of UFA recognition using different feature factors are compared, and the introduction of features is proven to be effective.

The remainder of this article is organized as follows. The study area and datasets used are described in Section 2. In Section 2.3, the methodology is explained. The experimental results and comparative analyses are presented in Section 3. Section 4 discusses the advantages and limitations of the research method and suggestions for future work. Finally, this study is summarized in Section 5.

## 2. Materials and Methods

### 2.1. Study Area

**Figure 1:**
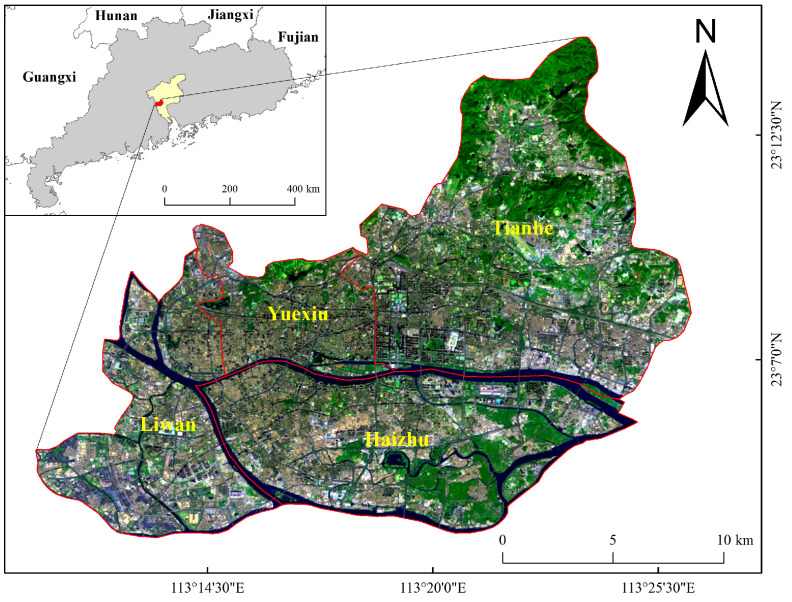
Area of study in the core area of Guangzhou, China. The remote sensing image is a Landsat 8 image obtained in 2020 with a spatial resolution of 30 m.

Guangzhou is the capital city of Guangdong Province, China. It is located at the mouth of the Pearl River and is the core of the Guangdong–Hong Kong–Macao Greater Bay Area. As of 2020, the city had 11 districts with a total area of 7434.4 km^2^, and its latitude and longitude range from 112°57′ to 114°3′ E and 22°26′ to 23°56′ N, with a permanent population of 18.67 million and an urbanization rate of 86.46%. The core urban areas of Guangzhou were selected for investigation, including the four municipal districts of Liwan, Yuexiu, Tianhe, and Haizhu (Figure 1), which are the most densely populated and economically active areas in the city. In recent years, Guangzhou has been expanding rapidly. To promote the healthy and sustainable development of Guangzhou, the reasonable planning of its UFAs should be considered. However, the urban planning maps designed by the government are difficult to update in time, so it is very important to map UFAs both accurately and promptly.

### 2.2. Data Sources

In this study, building footprints, POI data, and remote sensing data were used to estimate and classify UFAs (Table 1). Building footprints in 2020 were collected from the Gaode Map API, which included a total of 88,922 buildings with height information, and the area and perimeter indicators of each building were calculated to construct relevant features. The POI data in 2020 were also collected from the Gaode Map API. Each record included the latitude, longitude, name, address, telephone number, POI category, and administrative district, and a total of 72,489 POIs were selected. UFAs with the blocks formed by the road network were recognized as the basic unit. The road network data were downloaded from OpenStreetMap (OSM). The Landsat 8 remote sensing images were obtained from the Geospatial Data Cloud website. The images have a spatial resolution of 30 m and were imaged on 18 February 2020. The images were pre-processed according to radiometric calibration and atmospheric correction methods recommended by the United States Geological Survey (USGS) (http://glovis.usgs.gov/ accessed on 11 November 2021). Street View imagery provided by Baidu Street View and high-resolution historical Earth imagery provided by Google Earth were used to assist in the collection and verification of the functional area samples. All data were reprojected to the Universal Transverse Mercator projection for area 49N.

### 2.3. Methodology

**Figure 2:**
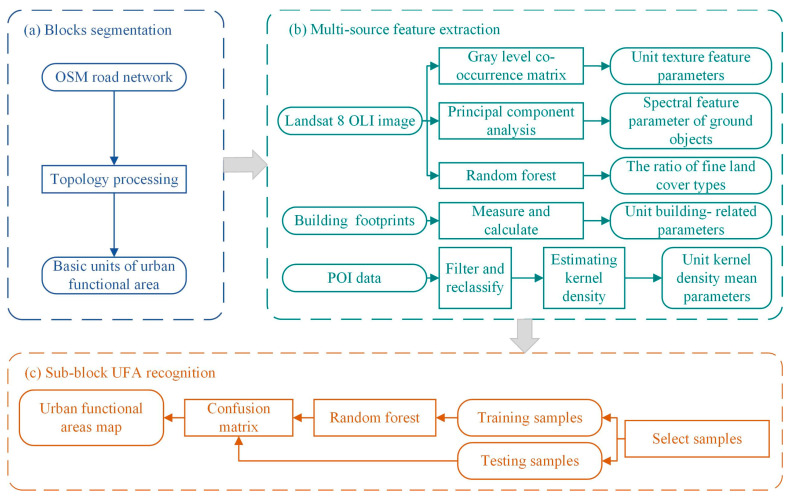
The framework for the recognition of UFAs.

This study consisted of the following three steps, as presented in Figure 2: (1) UFA segmentation; based on topology processing, the revised road vector was used to segment the basic spatial unit of urban function classification; (2) multi-feature extraction from multi-source data; (3) sub-block UFA recognition.

#### 2.3.1. Definition of UFA Types

The definition of UFAs is based on population mobility and social service functions. Five functional types of urban areas are defined in this study: residence, commercial, industry, public service, and green space. A block with a pure function is defined as a single-function area. In addition, there are some blocks with multiple functional uses simultaneously, which are divided into the following three main types: (1) mixed-function areas with a certain function as the dominant function, but their service effect and influence are far weaker than those of single-function areas; (2) mixed-function areas with multiple dominant functions that coexist obviously and have social functions with multiple functions; (3) mixed-function areas whose functions are not dominant and that have multiple functions simultaneously, but their various functions are not prominent.

#### 2.3.2. Blocks Segmentation

The block is the basic unit that undertakes socioeconomic functions in urban management and urban planning [34]. Thus, research on the block scale is of great significance for urban cognition. The city was divided into blocks by the urban road network, which served as the basic units for the recognition of UFAs. However, road network vectors obtained from OSM are characterized by the problem of hanging points or independent roads, which, when they are directly used to partition the study area, will lead to internal incompleteness and discontinuity. Thus, it is necessary to deal with topological errors in the road network to form polygonal closed cells bounded by road networks. Based on this, the division of UFAs can be realized.

To cull the road space without compromising the building footprint, reference was made to the method of Wang et al. [35], and the following processing was performed on the OSM data: (1) for roads with suspension points, the originally unconnected roads were connected; (2) for independent roads, the deletion process was performed; (3) after eliminating topological errors, the reclassification and hierarchical buffer processing was performed for all categories of roads in OSM (Table 2). After eliminating the road space, the blocks bounded by roads were formed. A total of 1925 blocks were obtained as the object of urban function recognition in this study.

#### 2.3.3. Extraction of Building Features

Buildings and blocks are closely related to each other. As the major sites of human activities, differences between the physical features of buildings can often reflect the functions of areas [22,36]. Many studies have confirmed that building features do have the potential to distinguish functional urban areas [37,38].

Commonly used building features include the area (A), perimeter (P), and structural ratio (SR) of a building. The area represents the floor area of the building, the perimeter represents the perimeter of the building, and the structural ratio is the ratio of the building’s perimeter to its area. There are differences in the building features of different functional areas. For commercial areas, the values of the building area, perimeter, and structural ratio within the block may vary greatly. For example, shopping centers have large footprints and complex shapes, but business offices have small footprints and regular rectangular shapes. However, buildings in residential areas tend to be highly consistent when they are compared in terms of their building area, perimeter, and structural ratio. Because of the high similarity of buildings within residential areas, the values of these features fluctuate less. The area, perimeter, and structural ratio of each building were calculated, and their sum (Sum), average (Avg), and standard deviation (SD) were determined based on the block; this helped to distinguish different types of functional areas, especially commercial and residential areas.

The floor (F) indicates the number of floors of a building, which is different from the two-dimensional (2D) features such as the area, perimeter, and structural ratio of a building. The introduction of this feature can reflect the difference in the 3D features of different buildings. Buildings of different heights often carry different social functions. For example, commercial office buildings are usually located in the city center and have higher floors; buildings in residential areas are usually relatively uniform in height; factories in industrial areas usually have lower building heights; and green space areas have fewer buildings with lower floor heights. Therefore, the total number of floors (Sum_F), the average number of floors (Avg_F), and the standard deviation of floors (SD_F) within a block were counted to distinguish different categories of functional areas.

In addition, the total number (TN) and density (D) of buildings in the block were calculated. The total number of buildings refers to the sum of all buildings in the block. The density of buildings refers to the ratio of the sum of the floor areas of buildings to the area of the block. Generally, residential areas have a dense distribution of buildings within them, and both these and commercial areas have a higher building density. In contrast, green space areas differ significantly from other areas in that both the density and the total number of buildings are relatively low. These two factors can reflect the distribution of buildings in the block to some degree.

**Figure 3:**
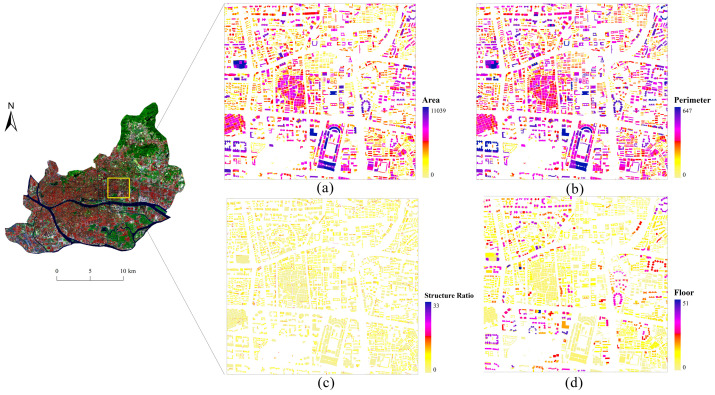
The thematic maps of building footprints: (**a**) building area; (**b**) building perimeter; (**c**) building structure ratio; (**d**) building floor.

A total of 14 related factors of these building features were obtained. First, the area, perimeter, and structure ratio of each building were calculated using ArcGIS (Figure 3). Then, using the spatial joining method to join the buildings into their blocks, the indicators, including Sum_A, Avg_A, SD_A, Sum_P, Avg_P, SD_P, Sum_SR, Avg_SR, SD_SR, Sum_F, Avg_F, SD_F, TN, and D, were obtained via statistical calculation to represent the building features of the block.

#### 2.3.4. Extraction of Socioeconomic Features

**Figure 4:**
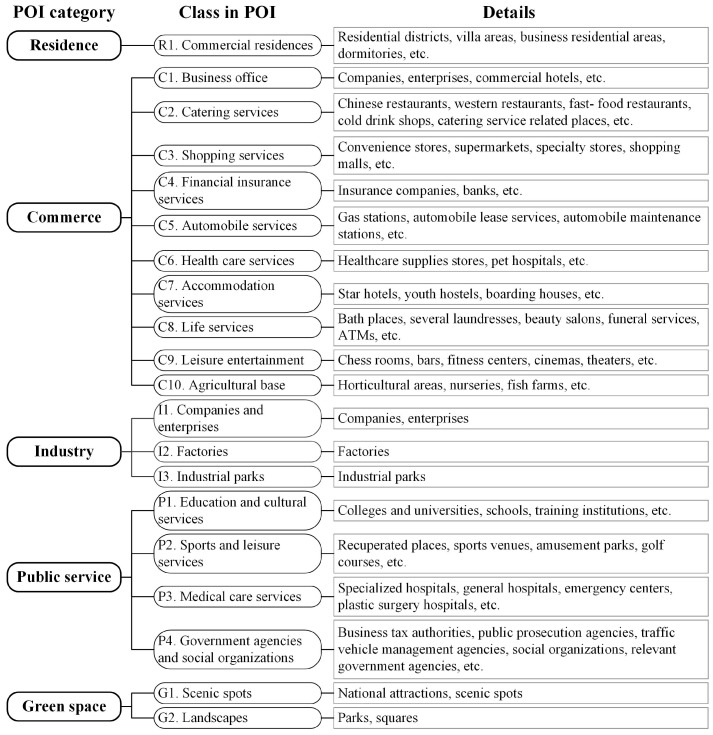
The POI reclassification details.

POIs are geographical points with category labels generated by human economic activities, and have been demonstrated to play an important role in functional area recognition [39]. The collected POIs contained 14 categories, including catering services, landscapes, companies and enterprises, shopping services, transportation facilities services, financial insurance services, education and cultural services, commercial residences, public facilities, life services, sports and leisure services, medical care services, government agencies and social organizations, and accommodation services. These categories are numerous and cross-repeat. Therefore, reclassification was needed for the initial POIs [40], and, before reclassification, points with low public awareness, such as bus stops, public toilets, and logistics and express delivery, were eliminated [41]. Because many road spaces were eliminated, reference was made to the POI classification criteria to reclassify them into a total of five categories: residence, commerce, industry, public service, and green space (Figure 4). It is worth noting that the company and enterprise POIs were not completely classified as industrial; the company and enterprise POIs located in buildings with more than seven floors were classified into the business office category of commercial functionality [22].

Because POIs are points abstracted from actual buildings, the area of a POI entity within a functional area has a significant impact on the function of that unit. Reference was made to the general area and scoring criteria of POIs [42], the results of street view images and online search surveys were combined, and corresponding weights were assigned to each POI category (Table 3).

**Figure 5:**
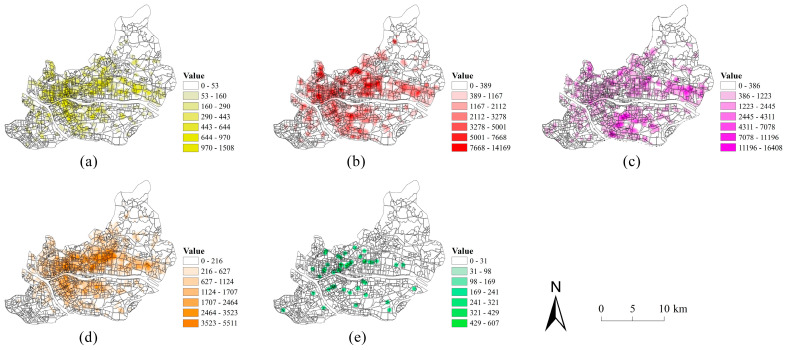
The kernel density estimation of different categories of POIs (**a**) residence; (**b**) commerce; (**c**) industry; (**d**) public service; (**e**) green space.

To realize the diffusion of the facility service influence of POIs and weaken the dispersion of POIs, the kernel density estimation method was used to analyze the POIs [43]. Related studies have shown that the kernel function has little effect on the kernel density estimation results, while the effect of the bandwidth is stronger [44]. By comparing the kernel density effects of different bandwidths (300 m, 500 m, 800 m, and 1000 m), 500 m was determined as the optimal bandwidth for this study. The kernel density estimation results of different categories of POIs are shown in Figure 5.

Then, with the spatial analysis tools of ArcGIS, the kernel density values of each unit in the five categories of POIs were obtained based on the blocks, and their proportions were calculated to represent the socioeconomic features of each block.

#### 2.3.5. Extracting Image Features

Remote sensing images can reflect the real condition of ground object distribution. Based on previous studies [31,45,46], spectral and texture features were used for recognition, and finer land cover features were introduced to enhance the recognition effect. First, radiometric calibration and atmospheric correction were performed on the Landsat 8 OLI images using ENVI software.

(1) The principal component eigenvalues obtained by principal component analysis of the images represent the spectral features of the ground objects [47,48]. The principal component analysis can be used to highlight image information, so that each band of image data has different information about the strength or weakness of the features. All the original band image data were selected for the principal component analysis transformation, and the desired information was enhanced and highlighted, while other information was suppressed. After principal component transformation, the difference of information in different bands was analyzed. Finally, the obtained first principal component (i.e., the transformed band 1) was determined to represent the spectral features of the image.

(2) The texture features of the images were described by the gray level co-occurrence matrix (GLCM) [46,49]. Eight Haralick texture measures [50], including the second moment, mean, homogeneity, entropy, dissimilar, correlation, contrast, and variance, were selected as the texture feature factors of the images. These can reflect the image grayscale information on the direction, adjacent interval, and change magnitude. Three key parameters are involved in the processing, i.e., window size, image element spacing, and gray level. After several experiments, the sliding window size of 3 × 3, the window shift distance of 1, and the quantized gray level of 64 were finally selected. After principal component transformation, band 1 contained the most information. Therefore, the transformed band 1 was used to calculate the texture features of the eight statistical factors.

(3) The finer land cover features of the study area are obtained by the coverage of ground objects. The results of coverage of ground objects are obtained by image mixing decomposition at a sub-pixel scale using the random forest model on pre-processed Landsat 8 OLI imagery [51]. The spatial resolution of this image is the same as Landsat 8 OLI. The accuracy of the classification results was verified using Google Earth high-resolution image data. The root mean square error (RMSE) was used to evaluate the classification accuracy of each ground object. The RMSE for all ground objects in 2020 was within 0.15. This method has high classification accuracy and can be used in the study of functional area recognition. In this study, the ground objects were finally reclassified into 9 classes: bare soil, woodland, grassland, water, shade, concrete, asphalt, metal, and other impervious surfaces. The coverage of the 9 ground object classes within each block was used to represent the finer land cover characteristics of the block.

In total, one spectral feature, eight texture features, and nine land cover features of the images were obtained. Based on the spatial analysis tools of ArcGIS, the spectral features, texture features, and finer land cover features of each block were, respectively, obtained.

#### 2.3.6. Sample Selection

**Figure 6:**
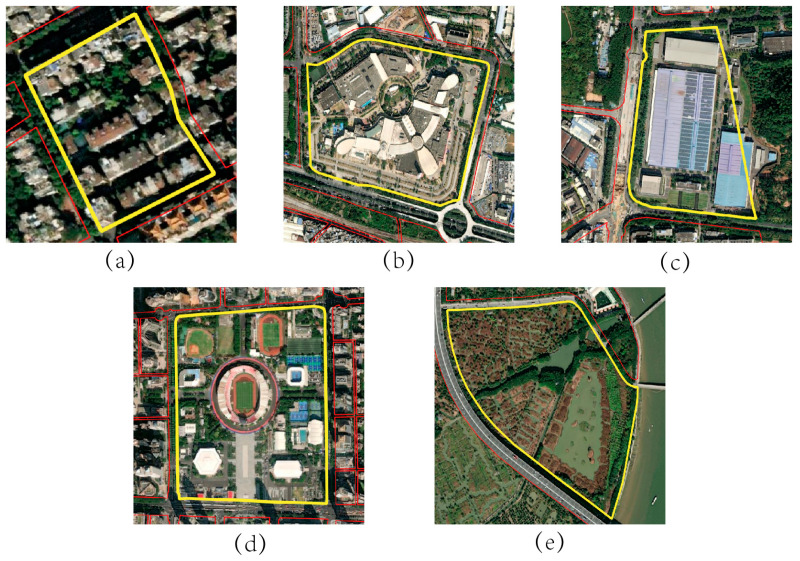
The diagrams of single-function area samples: (**a**) residential area (R) sample; (**b**) commercial area (C) sample; (**c**) industrial area (I) sample; (**d**) public service area (P) sample; (**e**) green space area (G) sample.

Samples were selected for the five types of single-function areas. First, the function of the blocks on the high-resolution images provided by Google Earth was confirmed. Fifty blocks were selected as samples for each of the five types of single-function areas, and were randomly divided into a training set and test set with a ratio of 4:1. Then, based on Baidu Street View, the sample categories were checked for the selected blocks. An example of a finalized single-function area is shown in Figure 6.

#### 2.3.7. Sub-Block-Level UFA Function Recognition

**Figure 7:**
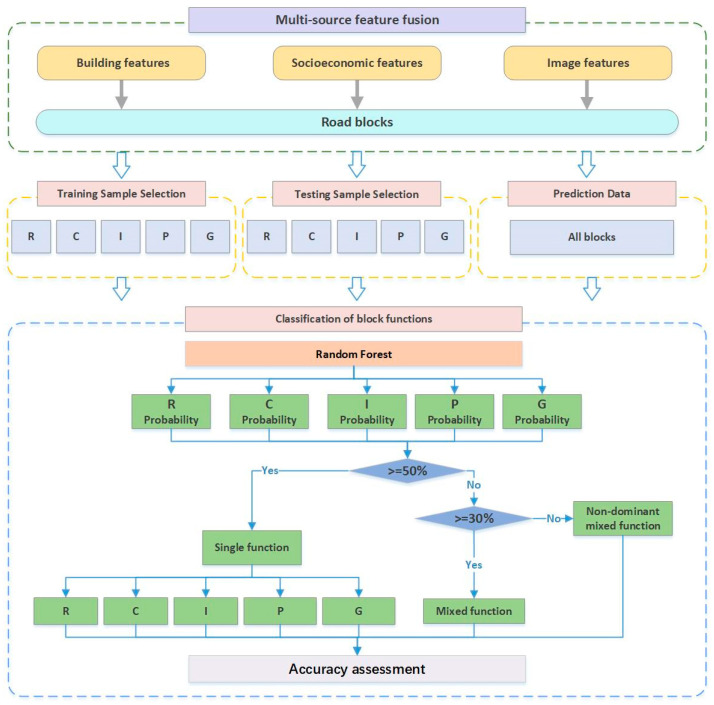
The flowchart of the sub-block-level UFA function recognition procedure.

Sub-block functional recognition is based on the random forest classifier. The random forest classifier has high prediction accuracy, high tolerance for outliers and missing values, less manual intervention, and very effective performance in classification tasks [52,53]. This study adopted the Random Forest Classifier from the sklearn for classification. The parameters of Random Forest Classifier from scikit-learn are set as: n_estimators = 50, max_depth = 10, and random_state = 42. By fusing 14 building features, 5 socioeconomic features, and 18 remote sensing image features, function classification at the sub-block level was achieved using a random forest classifier with blocks as the basic units. The classification details are exhibited in Figure 7. The model analyzes the architectural features, socioeconomic features, and remote sensing image features of the five types of single-function areas, and outputs the probability that each block is predicted as the five types of single-function areas.

The probability ratio of 50% is used as the criterion for judging the functional mixture of a block. When the probability of a block assigned to a certain function reaches or exceeds 50%, the block is determined to be a single-function area; when the probability of all functions does not reach 50%, the block is determined to be a mixed-function area. The mixed type also depends on the probability that the block is assigned to one of the five functional categories. When the probability of a block being assigned to a certain function reaches 30%, it means that the block has this type of dominant function. When the probability of a block being assigned to all five types of functional areas is less than 30%, it means that the block is a mixed-function area without dominant functions.

The accuracy of the recognition results is evaluated in terms of both single- and mixed-function areas, respectively. For single-function areas, the accuracy and reliability of the classification results are determined by using the confusion matrix. For mixed-function areas, the accuracy of the recognition results is verified by random sampling and visual comparison with the assistance of Google Earth’s high-resolution images and Baidu Street View.

#### 2.3.8. Comparing the Recognition Effects of Different Features

The socioeconomic features extracted from POI data, the morphological features and 3D features extracted from building footprints, and the spectral, textural, and finer land cover features extracted from remote sensing images all contribute to different degrees to the recognition results of UFAs. To emphasize the advantages of data features in this study, several sets of comparison experiments were conducted based on different features, including experiments on single-source features such as POI features (POI, P), building features (Building, B), and image features (Image, I), and experiments on combined features such as POI features and building features (POI + Building, P + B), POI features and image features (POI + Image, P + I), and building features and image features (Building + Image, B + I). Moreover, to highlight the contribution of 3D building features and finer land cover features to the optimization of functional area recognition, an experiment was conducted in which 3D building features were not introduced to Building2 (B2) for building features, an experiment in which finer land cover features were not introduced to Image2 (I2) for image features, and an experiment was conducted in which 3D building features and fine land cover features were not introduced to POI+Building2 + Imang2 (P + B2 + I2) for combined features.

A total of nine parallel experiments with different features were set up, and the recognition effects achieved using the combined POI features, building features, and image features (POI + Building + Image, P + B + I) used in this study were compared. The same recognition procedure as that used in this study was used to complete sub-block-level UFA function recognition.

## 3. Results

### 3.1. Recognition Results of UFAs

**Figure 8:**
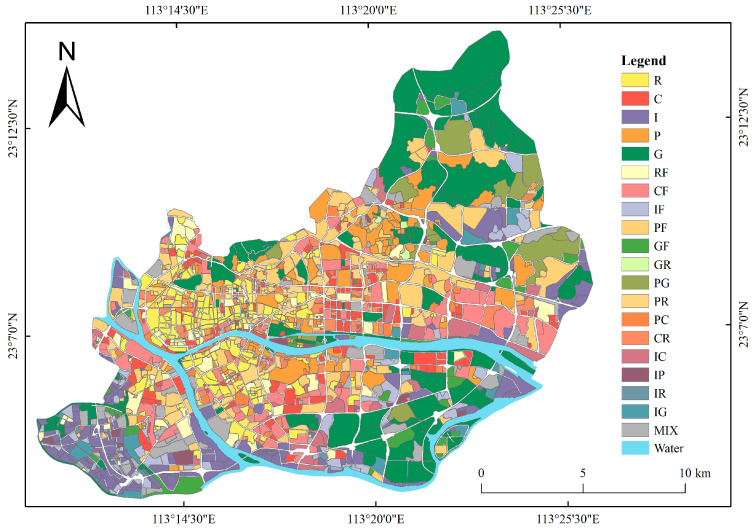
The recognition results of UFAs. (R: residential area; C: commercial area; I: industrial area; P: public service area; G: green space area; RF: mixed-function area dominated by residential use; CF: mixed-function area dominated by commercial use; IF: mixed-function area dominated by industrial use; PF: mixed-function area dominated by public service use; GF: mixed-function area dominated by green space use; GR: mixed-function area of green space and residential use; PG: mixed-function area of public service and green space use; PR: mixed-function area of public service and residential use; PC: mixed-function area of public service and commercial use; CR: mixed-function area of commercial and residential use; IC: mixed-function area of industrial and commercial use; IP: mixed-function area of industrial and public service use; IR: mixed-function area of industrial and residential use; IG: mixed-function area of industrial and green space use; MIX: mixed area without a dominant function.).

According to the research procedure described in Section 2.3.7, the results of urban functional zoning in the study area were obtained (Figure 8). There were 875 blocks recognized as single-function areas, accounting for 50.98% of the study area. Moreover, 1050 blocks were recognized as mixed-function areas, accounting for 49.02% of the study area.

It is evident from Figure 8 that the residential areas are mainly distributed in the Xiguan region of Liwan District and the northeast area of Haizhu District. The commercial areas are mainly located in the core of the study area such as Pearl River New Town and Canton Fair. The industrial areas are mainly concentrated in the southern area of Liwan District and the eastern area of the central part of Tianhe District, and the southern and eastern areas of Haizhu District are also slightly distributed. Public service areas are relatively evenly distributed throughout the study area, and are mainly concentrated in Tianhe District due to the number of universities located in the region. Green space areas are mainly distributed in Tianluhu Forestry Park, Guangzhou Haizhu National Wetland Park, Xiaoguwei Island Island, and other peripheral areas of the research area. The recognition results of single-function areas revealed an overall accuracy (OA) of 82% and a kappa coefficient of 0.78.

**Figure 9:**
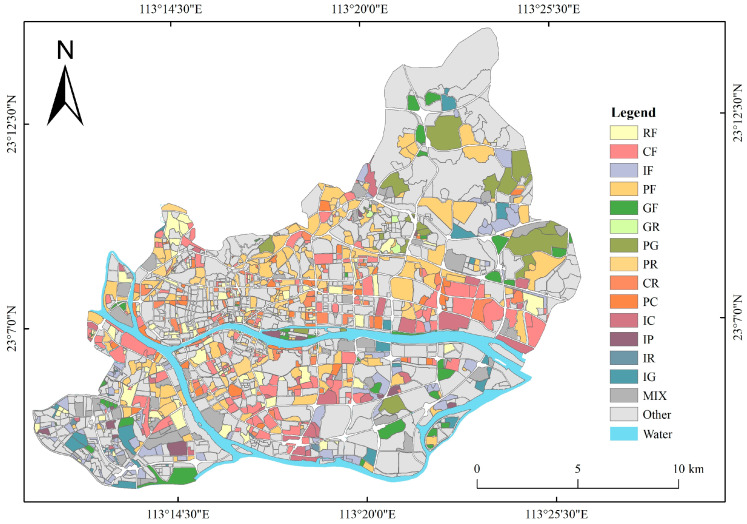
The recognition results of mixed-function areas.

The blocks assigned as mixed-function areas in the results were selected to obtain Figure 9. It was found that the mixed-function areas including residential, public service, and commercial functions were evenly distributed in the study area, which was consistent with the land-use information presented on the electronic map. By combining Google Earth and Baidu Street View, the recognition results of mixed-function areas in this study were further verified by random sampling, and the verification procedure is presented in Table 4.

According to two accuracy verifications, it was found that the recognition performance of residential and commercial areas was excellent; in particular, the relatively concentrated residential areas and main commercial centers in the study area were sufficiently identified. Many of the industrial areas could also be recognized, but there were industrial areas under construction with bare soil surfaces that may have been misclassified as green space areas. The recognition performance of the green space function in single-function areas was excellent, but the attributes of small parks or squares in mixed-function areas were not recognized. The results for public service areas were similar. The recognition accuracy of complete blocks formed by large schools and gymnasiums in the study area was high, but that of small public service facilities such as hospitals and kindergartens was poor, as these facilities are mostly located in mixed-function areas.

### 3.2. Comparison of the Recognition Effects with Different Feature Factors

#### 3.2.1. Comparison of the Classification Accuracy of Single-Function Areas

Parallel experiments were set up according to Section 2.3.8, and the recognition results of different features were obtained. The classification accuracy of single-function areas was verified by combining the test samples in this study, and the results are reported in Table 5. In parallel experiments using single-source features, it was found that Image (I) had the highest classification accuracy, and Building2 (B2) without 3D features had the lowest classification accuracy. In the parallel experiment of combining features, the fusion of the three types of features had the highest classification accuracy, the combination of POI + Building (P + B) had the lowest classification accuracy, and the difference in the classification accuracy between the two reached 30%.

The introduction of 3D building features and finer land cover features was found to be helpful for improving the accuracy of the recognition results. In the experiment with building features, the OA of Building (B) was 50%, that of Building2 (B2) was 38%, and the OA increased by 12% after the introduction of 3D building features. In the experiment with image features, the OA of Image (I) was 64%, that of Image2 (I2) was 50%, and the OA increased by 14% after introducing the finer land cover features. In the experiment with three types of data features, the OA of POI + Building + Image (P + B + I) was 82%, that of POI + Building2 + Image2 (P + B2 + I2) was 74%, and the OA increased by 8% after introducing the 3D building features and fine land cover features.

#### 3.2.2. Comparison of the Classification Accuracy of Mixed-Function Areas

**Figure 10:**
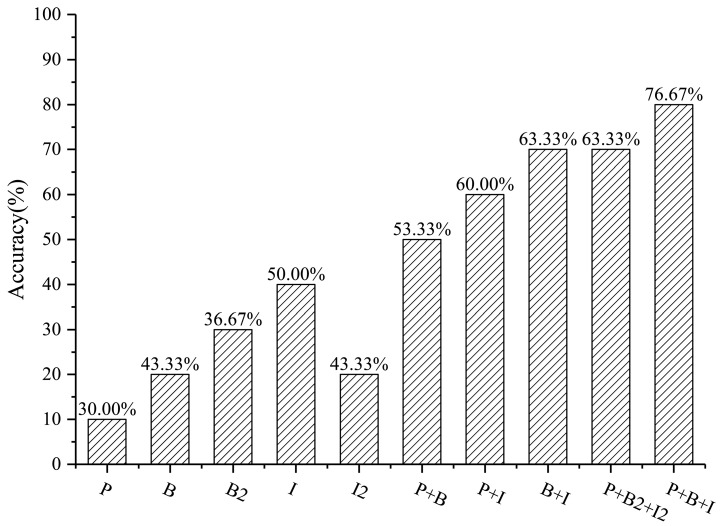
The classification accuracy of mixed-function areas with different features.

For the classification accuracy of mixed-function areas, 30 blocks assigned as having mixed functions were randomly selected to verify their recognition results. The classification accuracy of results with different features according to the number of statistics is shown in Figure 10.

**Figure 11:**
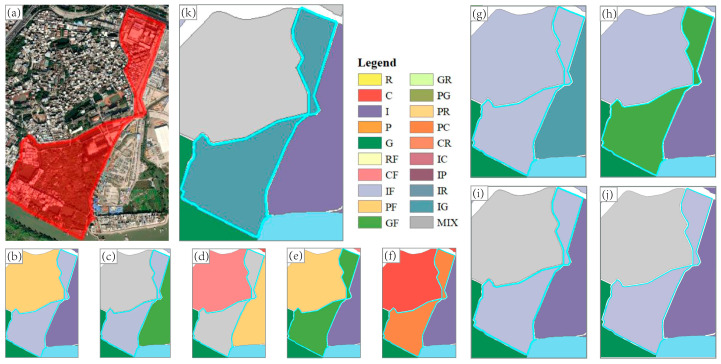
The comparison of the recognition results of different features for specific location 1. (**a**): the high-resolution imagery of Google Earth; (**b**): the result of P experiment; (**c**): the result of B experiment; (**d**): the result of B2 experiment; (**e**): the result of I experiment; (**f**): the result of I2 experiment; (**g**): the result of P + B experiment; (**h**): the result of P+I experiment; (**i**): the result of B + I experiment; (**j**): the result of P + B2 + I2 experiment; (**k**): the result of P + B + I experiment.

**Figure 12:**
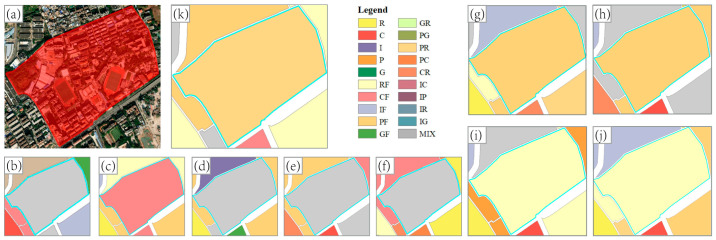
The comparison of the recognition results of different features for specific location 2. (**a**): the high-resolution imagery of Google Earth; (**b**): the result of P experiment; (**c**): the result of B experiment; (**d**): the result of B2 experiment; (**e**): the result of I experiment; (**f**): the result of I2 experiment; (**g**): the result of P + B experiment; (**h**): the result of P+I experiment; (**i**): the result of B + I experiment; (**j**): the result of P + B2 + I2 experiment; (**k**): the result of P + B + I experiment.

The classification accuracies of mixed-function areas were similar to those of single-function areas. The classification accuracy of Image (I) was still the highest among the single-source feature experiments, and the difference from the lowest classification accuracy was 20%. In the parallel experiment in which features were combined, the classification accuracy of the proposed method was still the highest, and reached 76.67%. Figure 11 and Figure 12 display two specific locations for comparison.

There is a large area of factory and green space in the block (Figure 11), which should have been recognized as a mixed-function area of industrial and green space (IG). However, in the experiment using single-source features, POI (P) recognized it as a mixed-function area dominated by industry. This is because there is a lack of POI labeling of green space, and the use of only POI features could not accurately extract the green space characteristics of the block. Building (B) and Building2 (B2), respectively, classified this block as an industrial-dominated mixed-function area and a mixed-function area without a dominant function. Building features can better classify a pure green space functional area. However, when the function of the block is mixed with other functions and green space, the recognition capability of building features for the green space function is very limited. Image (I) and Image2 (I2), respectively, recognized this block as a mixed-function area dominated by green space and a mixed-function area of public service and commercial use, and failed to correctly recognize the industrial function of the block. In the experiments using the combined features, POI + Image (P + I), POI + Building (P + B) and POI + Building2 + Image2 (P + B2 + I2) recognized the block as a mixed-function area dominated by industrial use, and Building + Image (B + I) recognized it as a mixed-function area dominated by green space. None of the four experiments resulted in the accurate extraction of the industrial and green space characteristics of the block, but the recognition results of the combination of POI + Building + Image (P + B + I) did achieve this. The block was designated as a mixed-function area of industrial and green space use, and the result was the most consistent with reality.

There is a large area of residential buildings and campus in the block (Figure 12), which should be recognized as a mixed-function area of public service and residence (PR). However, in the experiments using single-source features, POI (P), Image (I), Image (I2), and Building (B2) recognized the block as a mixed-function area without a dominant function and did not extract the characteristics of public service or residence, and the recognition effects were poor. Building (B) mistakenly recognized the block as a mixed-function area dominated by commercial use, which may have been due to the large differences between the floors and morphology of the residential buildings and campus buildings in this block. In the experiment using the combined data features, Building + Image (B + I) and POI + Building2 + Image2 (P + B2 + I2) recognized the block as a mixed-function area dominated by residential use, and failed to correctly extract the public service characteristics of the block. Both POI + Building (P + B) and POI + Image (P + I) recognized the block as a mixed-function area dominated by public service use, and failed to correctly extract the residence characteristics of the block. However, POI + Building + Image (P + B + I) recognized the block as a mixed-function area of public service and residential use, which was in line with the survey results.

On the whole, the classification accuracy of single-function areas was found to be higher than that of mixed-function areas, the accuracy of combined feature experiments was better than that of single-source feature experiments, and the recognition effect of the proposed method was better than that achieved in other parallel experiments. The comparison with the classification accuracy of parallel experiments also effectively proves that the introduction of 3D building features and finer land cover features was helpful for improving the recognition effect of UFAs.

## 4. Discussion

In this study, open access urban multi-source data were used to classify area functions at the urban sub-block level, and a solution with easily accessible data, easy-to-implement methods, and more comprehensive classification results was proposed. Previously, some scholars integrated multi-source data and used machine learning models to classify UFAs, but did not analyze regions in urban areas with mixed functions [16,54,55]; they only classified UFAs into four or six single-function categories based on different data feature information. The present research differs from these previous studies in that the classifications of functional areas were refined, and the results achieved reliable accuracy. Fine urban functional zoning not only promotes understanding of local conditions, but also allows quantitative analysis of spatial differences among different functional areas within cities. The recognition of green space and related sub-classes is of great research significance for studying ecosystem services and mitigating the urban heat island effect [56]. The recognition of public services and commercial and related sub-classes has positive significance for urban facilities construction improvement and hot spot detection [57,58]. In addition, the distribution of fine functional areas has certain reference value for the analysis of the causes of traffic congestion [59], the evaluation of regional functional use intensity [23], and the planning calibration [60] and renewal of cities [61]. Some scholars have also finely classified UFAs, but the data sources were different [5,23,62]. Other studies have made a finer classification of UFAs based on POI data, but this method only determines the mixed functions of the area via the frequency density and category ratio of POIs, which is not sufficient for the comprehensive consideration of the urban environment [35,63,64]. Some studies integrated multi-source data for the finer classification of UFAs and achieved reliable accuracy. For example, Liu et al. [44] fused landscape features of images, human activities based on taxi trajectory data, and functional information of POIs. Shannon’s information entropy [65] was used to measure the mixed degree of functional categories for an area. However, the taxi trajectory data used in the study were not open access, thereby limiting its wide suitability.

This study achieved the finer function classification of UFAs by fusing multi-features of open access data. The main challenges and potential improvements in this study are as follows.

(1) The introduced finer land cover features are reliable. Land cover features are closely related to the functional category of the block, and the finer land cover features introduced in this study were obtained based on medium-resolution data using a random forest model. The prerequisite for accurate classification is the need to ensure that the selected samples are pure. Reliable land cover classification results were obtained in this study using images with a spatial resolution of 30 m, but there was still a certain degree of error, and this error may affect the utility of finer land cover features in the recognition of UFAs. To minimize the accumulation of classification errors, the use of images with a higher spatial resolution (higher than 30 m) [51] or the construction of a bidirectional iterative self-optimizing classification model [33] will be considered in the future to optimize the accuracy of finer land cover.

(2) Appropriate training samples were selected for the recognition of functional areas. To ensure the effectiveness of model training, the selected training samples must be typical. However, in fact, the number of blocks in urban areas with an almost pure function that can be used as training samples is very rare. In this work, samples were selected by the manual visual method and investigation, and it was difficult to obtain a large sample size. Additionally, because of the difficulty of directly visually determining the degree of the functional mix of blocks, there were no samples selected for the training and classification of the mixed-function areas. Instead, according to the random forest classification, each block was assigned as different functional areas based on probability ratios to determine the category of the mixed-function area. Finally, high-resolution maps and street views were used to verify the recognition results. Moreover, as a sufficient number of blocks that function as pure transportation could not be selected, to ensure the classification accuracy of the model, the categories of functional areas defined in this study did not include transportation areas. This may have led to blocks such as railway stations being recognized as having industrial or commercial functions, or as a mixture of both because of their building morphology, material, and land cover composition; however, according to the functional definition of this study, they should have been recognized as a public service area. To obtain a larger sample size and allow the model to achieve a better recognition effect, a follow-up study will consider labeling the samples and verifying the results by integrating other data sources, such as land-use data. This can not only ensure the sample size, but can also improve the scientificity of the sample selection and result verification. However, these data of government departments are not publicly available at present, and the use of land-use data obtained via image supervision classification may lead to more errors. In addition, due to the differences in the surface landscapes of different cities, it is necessary to re-select samples for land cover and functional areas during migration application.

(3) The finer and more accurate mapping of UFAs can provide a scientific basis for urban macro-decisions, public service, and “smart city” construction. At present, the recognition results of this framework have achieved reliable accuracy, but there remains room for further optimization. On the one hand, the data used in this study were all open access data, and the quality and information contained in these data are often lower than those of commercial data. In the future, commercial data with higher spatial and temporal resolution or richer socioeconomic characteristics will be used to obtain more accurate recognition results. On the other hand, the organic integration of model parameters was performed. The 37 feature factors introduced in this study were not screened, which tends to lead to the redundancy of parameters and thus affects the accuracy of the recognition results. In a more detailed study in the future, the contribution of each factor to the classification of different functional areas will be estimated. Based on this, a hierarchical recognition framework will be constructed, and the imported parameters will be screened in a hierarchical manner to remove redundancy and achieve the organic integration of multiple features.

## 5. Conclusions

In this study, the multi-feature information extracted from building footprints, POI data, and Landsat images was combined, and sub-block-level urban function recognition was realized by using a random forest classifier. The conclusions are shown as follows:

(1) The recognition results of UFAs in the core urban area of Guangzhou, China showed that the area of single-function areas was slightly larger than that of mixed-function areas. Moreover, the OA of single-function areas was 82%, and the recognition accuracy of mixed-function areas was 76.67%.

(2) The comparison of recognition performance of parallel experiments showed that the introduction of 3D building features and finer land cover features was helpful for improving the recognition accuracy of UFAs. The OA of single-function areas increased by 8%, and the recognition accuracy of mixed-function areas increased by 13.34%.

The strength of this study is the method of the finer classification of UFAs using an easy-to-realize random forest classifier via open access multi-source data, which has wide applicability. Furthermore, the obtained research results provide detailed functional information about urban areas, which is of great value in urban research and can be used to assist urban planning [66], population density estimation [67], and land surface temperature surveys [68].

## Figures and Tables

**Table 1:** The data sources.

Data Type	Function	Sources
Building footprint	Extract the building features of the block	Gaode map (https://lbs.amap.com/api accessed on 11 October 2021)
POI	Extract the social and economic characteristics of the block	Gaode map (https://lbs.amap.com/api accessed on 25 May 2021)
Road network	The basic data of blocks	OpenStreetMap (http://www.openstreepmap.org accessed on 26 April 2021)
Landsat 8 OLI image	Extract the physical characteristics of the block	Geospatial Data Cloud (http://www.gscloud.cn/sources accessed on 11 November 2021)
Street View	Assistance for sample collection and verification	Baidu Street View (https://map.baidu.com/ accessed on 10 August 2022)
Google Earth	Assistance for sample collection and verification	Google Earth (https://earth.google.com/web/ accessed on 10 August 2022)

**Table 2:** The buffer processing of the OSM road network.

Road Buffer Level	Class in OSM
First buffer (20 m)	motorway, motorway_link, tertiary, tertiary_link, track, trunk, trunk_link
Second buffer (10 m)	secondary, secondary_link
Third buffer (5 m)	living_street, path, primary, primary_link, residential, service, unclassified, unknown

**Table 3:** The POI area weight assignments.

Area (m²)	POI Class	Score (Points)
<500	C2, C3, C6, C8, C9	3
500–1000	C1, C4, C5, C7, I1, P2, P4	8
1000–5000	C10, I2, P1, P3, R1	40
5000–10,000	G1, G2	80
≥10,000	I3	100

**Table 4:** The comparison of the partial functional block classification results.

Contrast Block	Google Map Image	Classification Result	Baidu Map Street View
A mixed-function area of green space and residential	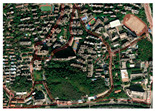	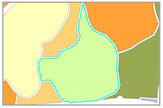	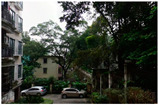
A mixed-function area of public service and commercial	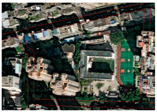	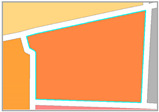	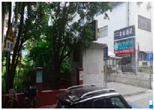
A mixed-function area of industrial and green space	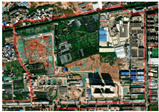	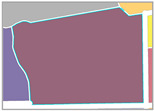	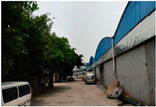
A mixed-function area of public service and residential	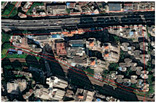	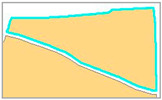	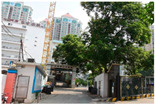
A mixed-function area of public service and green space	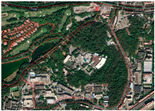	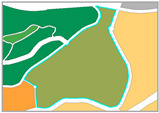	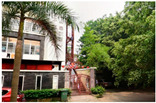
A mixed-function area of industrial and commercial	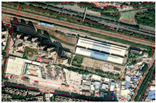	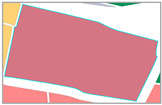	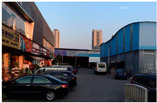
Legend	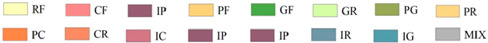		

**Table 5:** The classification accuracy of single-function areas with different feature factors.

Input Feature Factors	Residential Area	Commercial Area	Industrial Area	Public Service Area	Green Space Area	Overall Accuracy
P	70%	30%	20%	50%	60%	46%
B	70%	40%	50%	0%	90%	50%
B2	40%	30%	20%	10%	90%	38%
I	50%	60%	90%	50%	90%	64%
I2	50%	60%	70%	20%	90%	50%
P + B	60%	40%	70%	20%	70%	52%
P + I	80%	70%	80%	50%	90%	74%
B + I	60%	70%	80%	60%	100%	74%
P + B2 + I2	60%	70%	90%	50%	100%	74%
P + B + I	100%	70%	90%	60%	100%	82%

## Data Availability

The data presented in this study are available on request from the corresponding website.
